# Skin Sensitization Potential and Cellular ROS-Induced Cytotoxicity of Silica Nanoparticles

**DOI:** 10.3390/nano11082140

**Published:** 2021-08-22

**Authors:** Sung-Hyun Kim, Dong Han Lee, SeoYoon Choi, Jun-Young Yang, Kikyung Jung, Jayoung Jeong, Jae Ho Oh, Jin Hee Lee

**Affiliations:** Division of Toxicological Research, National Institute of Food and Drug Safety Evaluation, Ministry of Food and Drug Safety, Osong, Cheongju 28159, Korea; donghan04@korea.kr (D.H.L.); sugar819@korea.kr (S.C.); yangjy@korea.kr (J.-Y.Y.); kikyung@korea.kr (K.J.); 0jjy@korea.kr (J.J.); chopin68@korea.kr (J.H.O.)

**Keywords:** silica, nanoparticles, skin sensitization, KeratinoSens^TM^, h-CLAT, LLNA, alternative test

## Abstract

Nowadays, various industries using nanomaterials are growing rapidly, and in particular, as the commercialization and use of nanomaterials increase in the cosmetic field, the possibility of exposure of nanomaterials to the skin of product producers and consumers is increasing. Due to the unique properties of nanomaterials with a very small size, they can act as hapten and induce immune responses and skin sensitization, so accurate identification of toxicity is required. Therefore, we selected silica nanomaterials used in various fields such as cosmetics and biomaterials and evaluated the skin sensitization potential step-by-step according to in-vitro and in-vivo alternative test methods. KeratinoSens^TM^ cells of modified keratinocyte and THP-1 cells mimicking dendritic-cells were treated with silica nanoparticles, and their potential for skin sensitization and cytotoxicity were evaluated, respectively. We also confirmed the sensitizing ability of silica nanoparticles in the auricle-lymph nodes of BALB/C mice by in-vivo analysis. As a result, silica nanoparticles showed high protein binding and reactive oxygen species (ROS) mediated cytotoxicity, but no significant observation of skin sensitization indicators was observed. Although more studies are needed to elucidate the mechanism of skin sensitization by nanomaterials, the results of this study showed that silica nanoparticles did not induce skin sensitization.

## 1. Introduction

Manufactured nanomaterials refer to materials that are created to have at least one cross-section of a size of 100 nm or less. The unique properties of nanomaterials are increasing their use value in various industries, including food and biomedical science fields. Silica nanomaterials are one of the major types of nanomaterials which are particularly used in the industrial and cosmetics fields [1]. Therefore, it is pivotal for the manufacturers and consumers who consume these substances to identify the silica nanotoxicity due to its potential for human exposure through various routes.

In recent years, to evaluate substances used in cosmetics, animal substitution test methods have been applied by reflecting the 3R (Replacement, Reduction and Refinement) principles for animals [2,3]. Specifically, the chemical and biological mechanisms related to skin sensitization such as allergic contact dermatitis have been summarized as the adverse outcome pathway (AOP) of skin sensitization [4]. The Organization for Economic Cooperation and Development (OECD) skin sensitization guidelines classify four key events: (1) molecular initiation event, (2), (3) cellular response (keratinocyte and dendritic cells) and (4) organ-level response based on the AOP-induced skin sensitization. However, since these alternative test methods are chemical-based test guidelines (TG), various studies are needed to apply them to nanomaterials.

Nanomaterials possess unique physicochemical properties that differ from other chemicals that form a major determinant of their toxicity potential [5,6]. Although nanoparticles (NPs) are minimally soluble under normal physiological conditions, some are soluble in certain media, such as lysosomal fluids and cause toxicity [7,8]. Fiber formal nanomaterials such as Carbon nanotubes (CNTs) induce cytotoxicity due to the piercing effect according to the shape of the particles [9,10]. In addition, due to their very small size, nanomaterials can bind to carrier proteins and induce an immunological response [11,12]. An accurate analysis of the physicochemical properties of nanomaterials is very important in identifying the major factors of toxicity caused by nanomaterials.

Traditional toxicity tests such as oral/inhalation have studied the toxicity of relatively various silica nanomaterials; however, relatively little information is known about their influences on the skin. Recently, several nanomaterials have been explored on the induction of skin sensitization. Moreover, the applicability of alternative animal testing methods has been evaluated for these analyses [13,14,15]. However, there is still a lack of toxicological information on skin sensitization induced by NPs. Therefore, this study aims to evaluate the skin sensitization potential of silica nanomaterials, which have high potential in the cosmetic field, by using animal alternative test methods based on the AOP in accordance with skin sensitization step by step.

## 2. Materials and Methods

### 2.1. Silica Nanoparticles

Silica (Silicon dioxide; SiO_2_, 10–20 nm) (NPs) was purchased from Sigma-Aldrich (St. Louis, MO, USA). Their primary size was confirmed by transmission electron microscopy (TEM; JEM-1200EX II, JEOL, Tokyo, Japan) and TEM average size was calculated by counting more than 30 individual NPs using the inbuilt software program. The hydrodynamic size and zeta potentials of the silica NP were measured by a Zetasizer-Nano ZS (Malvern, Malvern Hills, UK). In addition, to verify that the NPs were not contaminated with endotoxins, the Endpoint Chromogenic Limulus Amoebocyte Lysate (LAL) assay (Cambrex, Walkersville, MD, USA) was performed. The solubility of silica NPs was measured in artificial lysosomal fluid (ALF, pH 5.5) as previously described [15,16]. Briefly, silica NPs were dispersed in each medium at 100 μg/mL and incubated for 48 h at room temperature. Centrifugation was performed thrice at 15,000× *g* for 60 min to collect the NP-free supernatant, and the absence of NPs was confirmed by dynamic light scattering (DLS) analysis using the Zetasizer Nano ZS. The concentration of ions in the supernatant was measured using the inductively coupled plasma optical emission spectroscopy (ICP-OES, 700-ES) (Varian, Palo Alto, CA, USA). Solubility was calculated into percentage of dissolved ion concentration regarding the initial mass of silica in the NP suspension.

### 2.2. Serum Protein Binding Affinity Test

Since there is a possibility that ‘haptenization’ of small molecules can act as a sensitizer, the binding ability of silica NPs with serum proteins was evaluated. Briefly, Silica NPs was dispersed in distilled water (DW) solution at concentration 1 mg/mL and incubated with 100 μg/mL bovine serum albumin (BSA) for 0, 4 and 24 h at room temperature. The samples reached at each time point were removed from the pellet by centrifugation at 15,000× *g* for 10 min, 3 times and the levels of protein in the supernatant were measured by using the bicinchoninic acid (BCA) assay (Thermo Fischer Scientific, Rockford, IL, USA). After the absorbance measurement, the amount of adsorbed BSA was calculated by subtracting the protein measurement value from the measurement value in the vehicle control group.

### 2.3. Preparation of Silica NPs Suspensions

Silica NPs suspensions were prepared by slightly modifying a formerly described method [15,17]. Briefly, silica NPs stock solutions were dispersed in DW and sonicated at 40 kHz with 100 W output power for 15 min in a bath-type sonicator (Saehan-Sonic, Seoul, Korea). Thereafter, fresh Dulbecco’s Modified Eagle’s Medium (DMEM) medium supplemented with 1% FBS was added to different working concentrations (0.98–2000 µM). In the human Cell Line Activation Test (h-CLAT) assay, silica NPs stock solution was dispersed in phosphate buffered saline (PBS) (pH 7.4) and sonicated at the same procedure. Fresh complete Roswell Park Memorial Institute-1640 (RPMI) medium supplemented with 10% FBS was added to different working concentrations (0–1200 µg/mL). Since the re-aggregation of silica NPs might be induced when the cell culture medium was added, ultrasonic dispersion was performed for additional 10 min. In the local lymph node assay (LLNA): 5-bromo-2′-deoxyuridine (BrdU)-flow cytometry (FCM) assay, silica NPs stock solution was dispersed in DW and sonicated at the same procedure. After that, 3% of the serum in the final volume was added to the dispersed stock solution and further dispersed for 10 min. Finally, as the re-aggregation of NPs might be induced when the DMF solution (working concentration: 25, 50, and 100% *v*/*v*) was added, ultrasonic dispersion was performed for additional 10 min.

### 2.4. KeratinoSens^TM^ Assay Test Methods

A transgenic human keratinocyte cell line with a stable insertion of the Luciferase reporter gene under the control of the Antioxidant Response Element (ARE)-element KeratinoSens^TM^ cells was provided by Givaudan Suisse SA (Vernier, Switzerland). The cells were cultured in DMEM media supplemented with 10% FBS, 0.5 mg/mL Geneticin (Sigma-Aldrich). The cells were sub-cultured every 2–4 days at 80–90% confluence for a maximum of 25 passages. The culture medium was replaced to a fresh medium and incubated in a humidified atmosphere condition of 5% CO_2_ at 37 °C. Stabilized KeratinoSens^TM^ cells were seeded into a 96-well cell culture plate at a density of 1 × 10^4^ cells/well and incubated overnight. The prepared cells were washed once with pre-warmed pH 7.4 Dulbecco’s phosphate buffered saline (DPBS), followed by the addition of dispersed silica NPs suspension (0.98–2000 μM), and the culture plates were then incubated for 48 h. Positive control, cinnamic aldehyde (CASRN. 14371-10-9, Sigma-Aldrich), was tested in parallel (concentration: 4–64 μM). The viability of KeratinoSens^TM^ cells was measured using the thiazolyl blue tetrazolium bromide (3-(4,5-dimethylthiazo-2-yl)-2,5-diphenyl-tetrazolium bromide (MTT) assay reduction test (Promega, Madison, WI, USA). To exclude the colorimetric interference from silica NP present in the cells, the supernatant was transferred into clear 96-well plates, and the absorbance was measured at 570 nm with a multi-microplate reader (Synergy HTX, BioTek, Winooski, VT, USA). The cell viability (%) was calculated based on the optical density (OD) of the vehicle control and blank control. Then, to measure the luciferase activity of silica NP, we used the One-Glo^TM^ Luciferase assay kit (Promega). The luciferase assay was conducted under the same conditions as the MTT assay. The luminescence intensity of each sample was measured using a multi-microplate reader. Levels of luciferase intensity were calculated based on the luminescence values of the vehicle control and blank control.

### 2.5. h-CLAT Assay Test Methods

The human monocytic leukemia cell line (THP-1) was purchased from American Type Culture Collection (ATCC) (Manassas, VA, USA). The THP-1 cells were cultured in RPMI-1640 containing 10% fetal bovine serum (FBS), 2 mM L-glutamine (Life Technologies, Grand Island, NY, USA), 100 IU/mL penicillin (Life Technologies), and 100 U/mL streptomycin (Life Technologies). The cells were sub-cultured every 3–4 days at 80–90% confluence. Then, culture medium was replaced to a fresh medium and incubated in humidified atmosphere condition of 5% CO_2_ at 37 °C. Stabilized THP-1 cells were seeded and dispersed to 1 × 10^6^ cells/well/0.5 mL in a 24-well culture plate and prepared by dispersing the prepared nanoparticle working solution (334.9–1200 µg/mL) and vehicle control. Additionally, positive control (2,4-dinitrochlorobenzene; DNCB 4 µg/mL) was treated in 0.5 mL volume. After incubation for 24 h in an incubator, they were transferred to Fluorescence-activated cell sorting (FACS) tubes and washed three times. Then, FITC-CD86 Ab (CAT#555657, BD Biosciences), FITC-CD54 Ab (CAT#353108, BioLegend), and mouse IgG1 (isotype control) (CAT#555748, BD Biosciences) were added and stained. Finally, FACS buffer 200 μL and propidium iodide (PI) (CAT#P4864, Sigma) 10 μL were added to the cells and analyzed using a BD FACS Calibur™ flow cytometer (BD Biosciences). The geometric mean fluorescence intensity (MFI) of CD86 and CD54 was calculated according to the OECD TG 442E, and the relative fluorescence intensity (RFI) was calculated based on the following formula presented in the guideline. According to the guidelines, the criteria for determining a skin sensitizer (at least twice in 3 tests, at any test concentration) are CD86 RFI ≥ 150% and cell viability ≥ 50% or CD54 RFI ≥ 200%, and cell viability ≥ 50%. It is classified as a sensitizing substance.

$$RFI=\frac{\mathrm{MFI}\;\mathrm{of}\;\mathrm{chemical}\;-\;\mathrm{treated}\;\mathrm{cells}\;-\;\mathrm{MFI}\;\mathrm{of}\;\mathrm{chemical}\;-\;\mathrm{treated}\;\mathrm{isotype}\;\mathrm{control}\;\mathrm{cells}\;\times \;100}{\mathrm{MFI}\;\mathrm{of}\;\mathrm{solvent}/\mathrm{vehicle}\;-\;\mathrm{treated}\;\mathrm{ctrl}\;\mathrm{cells}\;-\;\mathrm{MFI}\;\mathrm{of}\;\mathrm{solvent}/\mathrm{vehicle}\;-\;\mathrm{treated}\;\mathrm{isotype}\;\mathrm{ctrl}\;\mathrm{cells}}$$

### 2.6. Intracellular Reactive Oxygen Species (ROS) Assay

To confirm the intracellular ROS generation in the KeratinoSens^TM^ and THP-1 cells exposed to silica NPs, the assay was performed according to the modified DCFDA/H2DCFDA-Cellular ROS Assay Kit (Abcam, Cambridge, MA, USA) protocol. Briefly, KeratinoSens^TM^ and THP-1 cells were prepared in a dark-96-well culture microplate with 1 × 10^4^ cells/well and 3 × 10^5^ cells/well, respectively. These cells were incubated with 1× DCFDA solution for 45 min at 37 °C in the dark and washed with pre-warmed DPBS to remove the DCFDA solution. Then, the dispersed silica NPs were treated for 24 h at 0.98–2000 μM (in KeratinoSens^TM^) and 334.9–1200 μg/mL (in THP-1). The fluorescence intensity induced by silica NPs was measured at 485/535 nm wavelength (λ) using a multi-microplate reader (Synergy HTX, BioTek, Seoul, Korea).

### 2.7. LLNA: BrdU-FCM Assay Test Methods

LLNA: BrdU-FCM assay was performed as previously described in Han et al.’s study [18] and based on recently revised OECD TG 442B (LLNA: BrdU-FCM) protocol. Female BALB/C mice (7 weeks old, Specific Pathogen Free; SPF) were purchased from KOATECH CO. (Pyoung-taek, Gyeonggi-do, Korea) and acclimated for at least 7 days before experiments. The animals were kept at an animal facility in the Korea Ministry of Food and Drug Safety (MFDS). The studies were approved by the Institutional Animal Care and Use Committee (IACUC) (Approval number: MFDS-20-013c2; Approval date: 23 April 2020). The animals were housed at a temperature of 22 ± 3 °C and relative humidity of 30–70%. The room was lit with artificial light for 12 h per day. Solid diets and sterilized drinking water were supplied ad libitum. Animals were randomly selected with body weight measurements.

During the first three days, dispersed silica NPs suspension (working concentration: 25, 50, and 100% *v*/*v*, respectively), vehicle control (DMF contained 3% mouse serum) and positive control (25% hexyl cinnamic aldehyde, HCA) were applied to the dorsal skin of each ear of the mouse at the same time-point (group per four). Acetone: olive oil (4:1 *v*/*v*, AOO) solvent was used to prepare the positive control based on the OECD TG 442B. Silica NPs suspension was prepared fresh daily before application. On day 5, all mice were intraperitoneally (i.p.) injected with 100 μL of BrdU solution (20 mg/mL). On day 6, mice were sacrificed, and their auricular lymph nodes were isolated. Excised lymph nodes were mashed using a spatula to prepare lymph node cells (LNCs). LNCs were counted using a hemocytometer after staining with trypan blue (Sigma). The quantitated LNCs (1.5 × 10^6^ cells/mL) were prepared for the LLNA: BrdU-FCM assay, via the protocol provided in the BD Pharmingen^TM^ FITC BrdU Flow Kit (BD Biosciences, San Jose, CA, USA). Viable LNCs were counted using a BD FACS Calibur^TM^ flow cytometer (BD Biosciences), and a total of 1 × 10^4^ gated cells were analyzed, as previously described [16,17]. The stimulation index (SI) was calculated using the formula described in the OECD TG 442B [12]. If the SI was 2.7 or greater, the test materials were classified as sensitizers.

$$Stimulation\;Index\;\left(SI\right)=\frac{\mathrm{Number}\;\mathrm{of}\;\mathrm{BrdU}\;-\;\mathrm{positive}\;\mathrm{LNCs}/\mathrm{mouse}\;\mathrm{exposed}\;\mathrm{to}\;a\;\mathrm{test}\;\mathrm{material}}{\mathrm{Mean}\;\mathrm{number}\;\mathrm{of}\;\mathrm{BrdU}\;-\;\mathrm{positive}\;\mathrm{LNCs}\;\mathrm{in}\;\mathrm{the}\;\mathrm{vehicle}\;\mathrm{control}\;\mathrm{group}}$$

### 2.8. Statistical Analysis

All data were analyzed with GraphPad Prism software ver. 5.0 (La Jolla, CA, USA) and expressed as mean ± standard error of the mean (SEM). Statistical analysis was performed by one-way ANOVA and each group was compared by post-hoc Turkey’s pairwise comparisons. Results with *p* values < 0.05 were considered statistically significant.

## 3. Results

### 3.1. Physicochemical Properties of the Silica NPs

**Figure 1:**
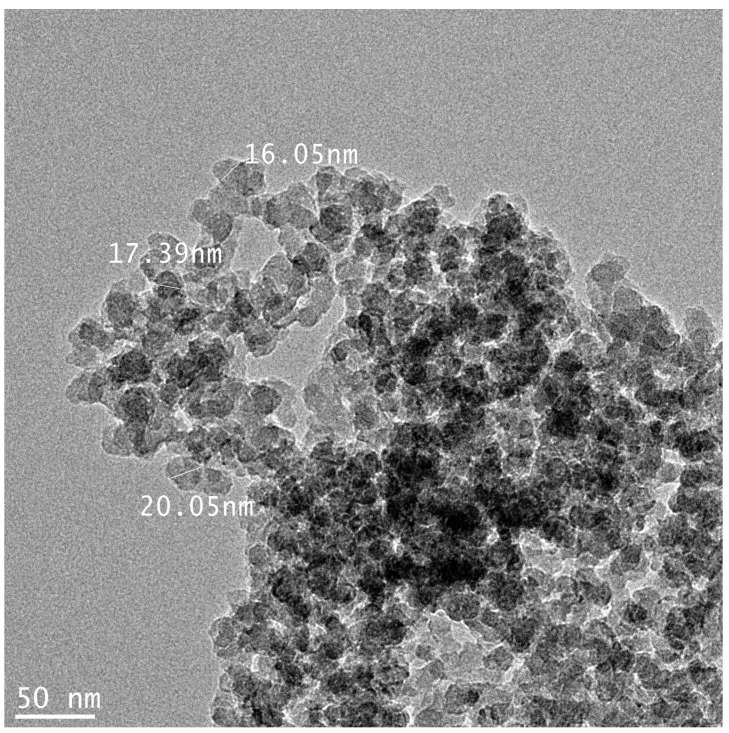
Morphological images of silica NP observed by transmission electron microscopy (TEM). (scale bar = 50 nm).

**Figure 2:**
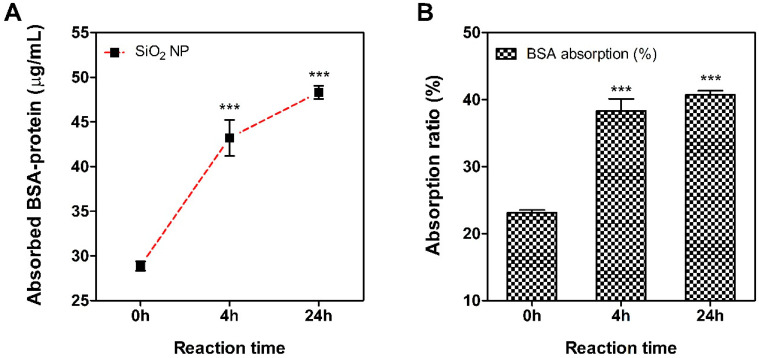
Protein binding affinity of silica nanoparticles using bovine serum albumin (BSA). After reaction with 100 μg/mL BSA, (**A**) Absorbed BSA protein amount, (**B**) protein absorption rate. Data are expressed as mean ± SEM (*n* = 8). *** *p* < 0.0001 compared to 0 h time-point.

Figure 1 shows the TEM images of silica NPs used in this study. TEM analysis results showed that silica NPs had a size of 15.56 ± 1.78 nm. The physicochemical characteristic of silica NPs is summarized in Table 1. The hydrodynamic size was observed to increase than the primary size when suspended in all types of medium. In particular, silica NPs showed a larger size in PBS solution than DW, and lower values were observed when prepared and dispersed as final working solutions for each test. Measurement of the zeta potential indicated that silica NPs were negatively charged in DW, PBS and final working solution. The solubility of nanomaterials in artificial lysosomal fluid (pH 5.5), the solubility was 0.02%. The silica NPs used in the test did not demonstrate contamination by endotoxin due to the LAL assay. In addition, since the reactivity between nanomaterials and proteins is crucial in the generation of hapten that can cause sensitization, the affinity between silica NPs and BSA protein was confirmed (Figure 2). After reacting the NPs with BSA (about 23%), it was bound to a level of 37% from 4 h and about 41% at 24 h.

### 3.2. Evaluation of Silica NPs in the KeratinoSens^TM^ Assay; Key Event II

**Figure 3:**
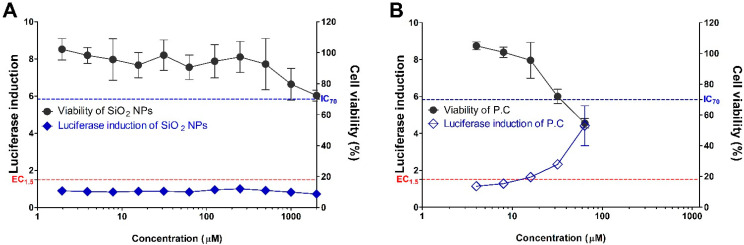
Induction of luciferase activity (blue line) and viability (black line) in the KeratinoSens^TM^ assay. The cells were treated with the (**A**) Silica NP, and (**B**) positive control (cinnamic aldehyde, CAS number 14371-10-9). Data are expressed as mean ± SEM (*n* = 6). Positive control was tested in parallel (4–64 µM).

The silica NPs were assessed for their skin sensitization potential using the KeratinoSens^TM^ assay (Figure 3). The cytotoxicity by the treatment of silica NPs showed a tendency to increase in a dose-dependent manner, and approximately 20–30% of cytotoxicity was induced at a high concentration of >500 μM or more among the treated silica NPs concentration group. IC50 values were found to be >2000 μM. Treatment with silica NPs did not induce luciferase activity in recombinant cells. The EC1.5 (interpolated concentration for a 1.5-fold luciferase induction) value for the silica NPs was >2000 μM, thus being classified as a non-sensitizer.

### 3.3. Evaluation of Silica NPs in the h-CLAT Assay; Key Event III

**Figure 4:**
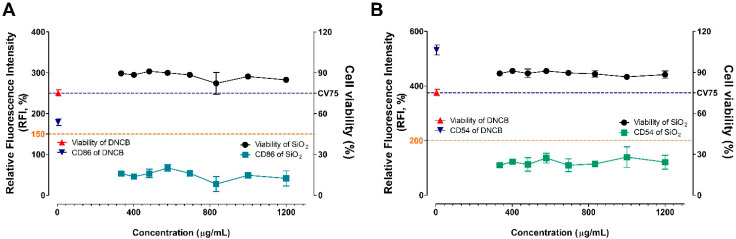
Evaluation of (**A**) CD86 and (**B**) CD54 expression, and cell viability in the h-CLAT assay. The cells were treated with the silica NP, and positive control (DNCB, CAS number 97-00-7). Data are expressed as mean ± SEM (*n* = 6). Positive control was tested *in parallel* (4 µg/mL). DNCB = 2,4-dinitrochlorobenzene.

The silica NPs were assessed for skin sensitization potential using the h-CLAT assay, which mimics the sensitizer response in dendritic cells (Figure 4). This test method quantifies the changes in surface proteins as markers of monocyte maturation. The cell viability was about 85–90% compared to the control group in the total concentration group of 334.9–1200 μg/mL, and dose dependence was not observed. Silica NPs did not induce the activity above the baseline in both CD86 (RFI = 150%) and CD54 (RFI = 200%), which were sensitive markers of the h-CLAT assay. Finally, silica NPs were judged as non-sensitizers because they did not meet the criteria for positive skin sensitizers of the h-CLAT guidelines.

### 3.4. Induction of Intracellular Reactive Oxygen Species (ROS) Following the Treatment of Silica NPs

**Figure 5:**
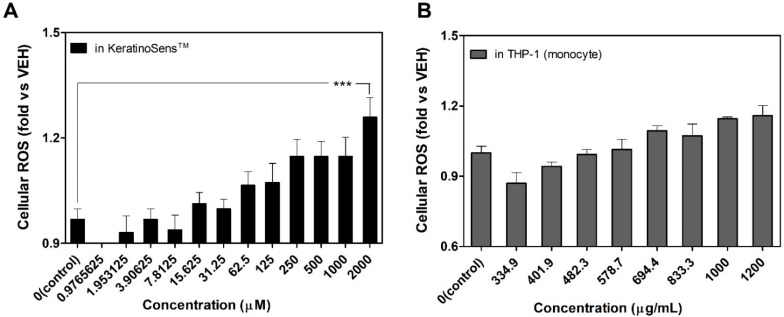
Evaluation of intra-cellular ROS generation treated with silica NPs. The levels of cellular ROS generated in (**A**) KeratinoSens^TM^ cells and (**B**) THP-1 cells. Data are expressed as mean ± SEM (*n* = 4). *** *p* < 0.0001 compared to vehicle control group. ROS = reactive oxygen species, VEH = vehicle control.

To elucidate the mechanism of silica NPs induced cytotoxicity, we assessed the intracellular ROS levels (Figure 5). Compared to vehicle control, treatment with silica NPs induced ROS production in KeratinoSens^TM^ cells and THP-1 cells 24 h after exposure. In addition, ROS generation in each cell was increased in a dose-dependent manner with silica NPs concentration, and statistical significance was observed especially at the highest concentration in KeratinoSens^TM^ cells.

### 3.5. Evaluation of Silica NPs in the LLNA: BrdU-FCM Assay; Key Event IV

**Figure 6:**
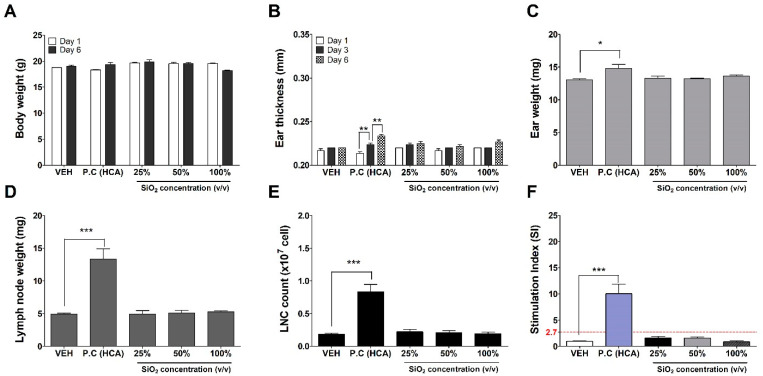
Results of silica NPs skin sensitization potential in LLNA: BrdU-FCM. The dispersed silica NP’s suspension (working concentration: 25, 50, and 100% *v*/*v*, respectively), vehicle control (DMF contained 3% mouse serum) and positive control (25% HCA) were applied to the dorsal skin of each ear of the mouse. The evaluation parameters were as follows: (**A**) Body weight (g), (**B**) Ear thickness (mm), (**C**) Ear weight (mg), (**D**) Lymph node weight (mg), (**E**) Number of lymph node cell (×10^7^ cells), and (**F**) Stimulation Index (SI). Data are expressed as mean ± SEM (*n* = 4). * *p* < 0.05, *** *p* < 0.0001 compared to vehicle control group (The ear thickness values were compared on days 1–3 and 3–6 after administration, ** *p* < 0.001). VEH = vehicle control, P.C = positive control, HCA = hexyl cinnamic aldehyde.

The silica NPs were assessed for their skin sensitization potential using the LLNA: BrdU-FCM, in vivo assay (Figure 6). No significant results were found at any concentration of silica NPs except for the positive control (25% hexyl cinnamic aldehyde) group for a total of 6 parameters: body weight, ear thickness and weight, lymph weight, number of lymph cell count, and stimulation index (SI) used for sensitization evaluation. Finally, the SI value was found to be less than 2.7, as calculated by flow cytometry, and was judged as a non-sensitizer through criteria of OECD TG 442B.

## 4. Discussion

Silica nanomaterials are promising materials for potential environmental and biomedical applications [19]. In particular, silica nanoparticles are attracting attention in the cosmetic field due to their low production cost and the characteristics of hydrophilic surface favoring protracted circulation [20]. However, the safety of human exposure to production workers (workplace) and product users can be ensured only by accurately identifying the toxicity of silica nanomaterials. The properties and cytotoxic effects of silica NPs have not been fully defined, and there is still controversy over their safety [21]. Therefore, it is crucial to identify the safety of the substance through various studies.

Recently, as the importance of the 3R test principle increases in the field of non-clinical toxicity testing related to cosmetics, the use of test methods that substitute animals has emerged as a major issue in the international community. Alternative test methods that do not involve the use of animals have been proposed by various countries and institutions, including the European Union Reference Laboratory for Alternatives to Animal Testing (EURL ECVAM), the Interagency Coordinating Committee on the Validation of Alternative Methods (ICCVAM), the Korea (KoCVAM) and the Japanese Center for Alternative Validation (JaCVAM). Research on this topic is currently underway and the OECD has approved, formulated and disseminated guidelines for alternative test methods. The OECD Skin Sensitization Alternative Testing Guidelines classify four major events: Key Event (1) Molecular initiation (protein reactivity), Key Event (2) and Key Event (3) Skin related cellular response (keratinocytes and dendritic cells), and Key Event (4) Lymph node level response (lymph node cell-differentiation) [22,23,24,25]. However, the AOP for skin sensitization as above has been applied based on soluble chemicals only.

Although nanomaterials are not soluble materials, their tiny size acts as a hapten and has a very high potential to become a sensitinogen. It is the initiation step of the sensitizer following haptenization that has a noticeable influence on the immunogenicity that induces sensitization [26,27]. We evaluated the affinity with the BSA protein as previously reported to confirm the hapten ability of the silica nanomaterial [28]. In addition to the methods we have measured, test methods for sensitive protein detection have been recently reported and can be used as a test method for protein adsorption of nanomaterials [29,30]. In this study, the results of the protein binding affinity of silica NPs were observed to easily react with the albumin protein present in a large number in the body, showing almost maximum reactivity in 4 h. This protein binding ability is a very important factor in the haptenization of sensitinogen [31]. Previous studies suggested that nanomaterials complexed with proteins can induce immune responses [32]. In addition, it has been reported that metal-based nanomaterials, including nickel, cobalt, copper and chromium, can also facilitate immune responses [33,34,35].

In our study, silica NPs were poorly soluble in vehicle solutions and characterized to be easily agglomerated. Since the dispersion process was important to predict the nanotoxicity accurately, we performed the task of uniformly dispersing the nanomaterials in the solutions. We used different serum proteins to disperse silica NPs in both the in-vitro KeratinoSens^TM^ test, the h-CLAT, and the in-vivo LLNA: BrdU-FCM assay. In the in-vitro test, the dispersion was induced by including FBS in the medium. Meanwhile, in the in-vivo test, inactivated mouse serum obtained in advance was used as a dispersing agent for nanomaterials [36]. This was chosen based on previous reports that inactivated sera obtained from the same species reduced hard aggregation and had no sera-induced side effects [37,38].

The size of nanomaterials in the final working solution of silica NPs for each test showed a soft-agglomerated hydrodynamic size compared to the primary size and ranged from approximately 295–467 nm (Figure 1 and Table 1). Approximately 0.5 μm in size is endocytosed through receptor independent endocytosis [39]. After endocytosis, it is hydrolyzed in lysosomes and then antigen can be presented by Antigen-presenting cells (APCs), so size is a very important factor [40]. In our study, the silica NP’s physical size in the working solution is considered to be the size that allows endocytosis. In general, phagocytosis by macrophages and dendritic cells can be strongly enhanced by cationic surface-charged particles of nanomaterials [41,42]. It is also known that, after endocytosis, small electron affinity substances such as sensitizers can act on the sensor protein keap-1 to activate the ARE- nuclear factor-erythroid 2-related factor 2 (Nrf2) signaling pathway [23]. In this study, it was observed that the surface charge was changed as the protein was adsorbed on the NPs surface. However, despite the induction of dispersion by the formation of the NPs-protein corona complex, the final immune response due to the immunogenicity and haptenization of the nanomaterials was not observed. These results indicate that silica NPs can adsorb proteins on the surface, but do not act as an immunogen after being phagocytosed by actual immune cells. Although further research is needed, in the current test, the effects on ions were excluded because silica NPs have very low solubility after intracellular phagocytosis. There was no close correlation between immunogenicity/sensitization and physicochemical effects of NPs themselves.

After confirming the high protein binding the ability of the silica NPs, the test outcomes for expression of the sensitization index in KeratinoSens^TM^ cells and THP-1 cells according to the AOP of skin sensitization procedure showed that the silica NPs were negative (non-sensitized). Although it was reported that the surface-modified silica nanomaterial promoted the expression of CD86, an activation marker, in h-CLAT, it was confirmed that the silica NPs used in our test did not show more than the standard value for the activation marker such as CD86 [43]. Although each cell treatment of silica nanomaterials induced some cytotoxicity, no significant increase in sensitization index was found.

In this study, no evidence for the skin sensitization of silica NPs was observed. Nonetheless, we found that the silica nanomaterials penetrated into cells and endogenous ROS generated, as in the results of intracellular ROS. Previous studies, including ours, have suggested that ROS generation by NPs was highly correlated with inflammatory or cytotoxic potential in vivo [44,45,46]. Specifically, silica nanomaterials have been reported to have ROS generating potential [47,48]. Therefore, based on these studies, it is suggested that silica-induced cytotoxicity in this study is one of the major factors by induction of endogenous ROS generated after phagocytosis.

The outcomes of in-vivo testing using silica NPs also showed negative (non-sensitizing) reactions. These findings were similar to a previous study evaluating silica NPs by LLNA testing [49]. Perhaps the aggregate formation of nanomaterials might not penetrate the barrier of the normal stratum corneum of animals [50]. Although further studies on skin permeation are likely to be needed, the treatment of silica nanomaterials in this study did not induce toxicological effects or skin sensitization on the skin of mouse ears.

The research findings we reported dealt with only one type of silica-derived nanomaterial. Since commercially available silica NPs can be manufactured in various sizes and shapes, to secure the safety of the Silica nanomaterials, it is suggested that additional toxicological data acquisition is necessary through more research. Moreover, it is necessary to protect workers in the workplace and establish guidelines for the skin sensitization specifically in terms of nanomaterials for use in various studies.

## 5. Conclusions

In conclusion, we reported the sensitization test results of silica NPs using the in-vitro and in-vivo alternative tests in the current study. We found that silica nanomaterials could induce some cytotoxicity upon ROS generation but did not induce skin sensitization in both in-vivo and in-vitro assays.

## Figures and Tables

**Table 1:** Physicochemical properties of silica NP.

Silica Nanoparticle (SiO^2^)	In Vitro Assay		In Vivo Assay
	In KeratinoSens^TM^	In h-CLAT	LLNA: BrdU-FCM
Primary size (nm)	10–20		
TEM average size (nm)	15.56 ± 1.78		
Hydrodynamic size (nm)			
in vehicle solution ^a^	432.9 ± 27.7	781.9 ± 118.5	306.0 ± 5.67
in working solution ^b^	467.8 ± 68.2	295.5 ± 4.3	304.0 ± 16.53
Polydispersity (PDI)			
in vehicle solution ^a^	0.47 ± 0.09	0.51 ± 0.11	0.31 ± 0.04
in working solution ^b^	0.54 ± 0.18	0.62 ± 0.03	0.37 ± 0.08
Zeta potential (mV)			
in vehicle solution ^a^	−0.43 ± 1.82	−35.50 ± 2.24	−0.16 ± 1.05
in working solution ^b^	−25.82 ± 1.20	−23.35 ± 2.03	−46.0 ± 1.62
Solubility (%) in ALF	0.02		
Molecular weight (g/mol)	60.1		
Purity (%)	99.5		
Endotoxin (EU/mL)	ND	ND	ND

^a^ DW (KeratinoSens^TM^ assay and LLNA:BrdU-FCM assay) and PBS (h-CLAT assay) were used as vehicle solutions. ^b^ Working solution (KeratinoSens^TM^ assay) were prepared with DW stock (1%) + DMEM, containing 1% FBS. Working solution (h-CLAT assay) was prepared with PBS stock (2%) + RPMI, containing 10% FBS. Working solution (LLNA: BrdU-FCM assay) was prepared using DW stock (10%) + DMF, containing 3% mouse serum. Data are expressed as mean ± SD, *n* = 6; DW = distilled water, DMEM = Dulbecco’s Modified Eagle’s Medium, FBS = Fetal bovine serum, RPMI = Roswell Park Memorial Institute-1640, DMF = N,N-Dimethylformamide, TEM = transmission electron microscopy, ALF = artificial lysosomal fluid, EU = endotoxin unit, ND = not determined.

## Data Availability

The original contributions presented in the study are included in the article, further inquiries can be directed to the corresponding authors.
